# New structures simultaneously harboring class 1 integron and IS*CR1-*linked resistance genes in multidrug-resistant Gram-negative bacteria

**DOI:** 10.1186/s12866-016-0683-x

**Published:** 2016-04-21

**Authors:** Cancan Cheng, Jingjing Sun, Fen Zheng, Wenting Lu, Qiu Yang, Yongyu Rui

**Affiliations:** Laboratory Medicine Center, Nanfang Hospital, Southern Medical University, Guangzhou, 510515 China

**Keywords:** Multidrug-resistant, Class 1 integron, IS*CR1*, Gram-negative bacteria

## Abstract

**Background:**

The connection structure of class 1 integron and insertion sequence common region 1 (IS*CR1*) is called “complex class 1 integrons” or “complex *sul1*-type integrons”, which is also known to be associated with many resistance genes. This structure is a powerful gene-capturing tool kit that can mobilize antibiotic resistance genes. In order to look for and study the structure among clinical multidrug-resistant (MDR) Gram-negative isolates, 63 isolates simultaneously harbored class 1 integron and IS*CR1*-linked resistance genes were isolated from 2309 clinical non-redundant MDR Gram-negative isolates in Nanfang Hospital in 2008–2013. The connecting regions between the class 1 integrons and IS*CR1* were examined using PCR and DNA sequencing to determine the structures in these isolates.

**Result:**

The two elements (the variable regions of the class 1 integron structures and the IS*CR1*-linked resistance genes) are connected in series among 63 isolates according to long-extension PCR and DNA sequencing. According to the kinds and permutations of resistance genes in the structure, 12 distinct types were identified, including 8 types that have never been described in any species. Several types of these structures are similar with the structures of other reports, but not entirely same.

**Conclusion:**

This study is the first to determine the structure simultaneously harboring class 1 integron and IS*CR1-*linked resistance genes by detecting the region connecting class 1 integrons and IS*CR1* in a large number of MDR bacteria. These structures carrying various resistance genes were closely associated with multidrug resistance bacteria in Southern China.

## Background

The increasing use of antimicrobial agents to treat Gram-negative bacterial infections has led to an increase in antibiotic resistance. Consequently, formerly routine therapies for many infectious diseases caused by multidrug-resistant (MDR) Gram-negative bacteria are now compromised. MDR bacteria evolve relatively quickly because the main driving force is lateral gene transfer, which is facilitated by a wide range of mobile genetic elements. The majority of these elements are integrons and transposons (including unit transposons and insertion sequences) [1]. Insertion sequences with common regions (IS*CR*s) are a type of insertion sequence.

In two previous studies, a total of 1329 and 1447 multidrug Gram-negative bacteria isolated in 2008–2009, was investigated for an ISCR1 [2] and a class 1 integron [3] respectively. In this study, 2309 clinical non-redundant MDR Gram-negative isolates were isolated between 2008 and 2013 at Nanfang Hospital, a 2200-bed tertiary-level teaching hospital in Guangzhou, China. Here, strains which carry a physical linkage between class 1 integrons and ISCR1 were focused. The IS*CR1* and class 1 integrons were characterized using PCR and DNA sequencing as the methods described previously [2, 3]. Of these strains, 63 isolates simultaneously harbored class 1 integrons and IS*CR1*-linked resistance genes were selected. The results were shown on the Table 1. Based on the results, the region connecting the IS*CR1* and the 3′-CS of the integron and the overall structures were investigated.

**Table 1:** Characterisation of complex class 1 integron in multidrug-resistant Gram-negative bacteria and resistance profiles of sequenced strains

Species	No. of isolates	Class 1 integron	ISCR	Type of complex class 1 integron
*Escherichia coli*	2	*aad*B-*aad*A2	*qnr*A1- *amp*R	B
	1	*dfr*A14-*arr*-2-*bla*_OXA-10_-*aad*A1	*bla*_DHA-1_-*amp*R	C
	1	*dfr*A25	*sap*A-like-*qnr*B2	D
	1	*aac*A4-*arr*-3-*dfr*A27- *aad*A16	short chain dehydrogenase/reductase- *qnr*B6	G
*Enterobacter cloacae*	2	*aad*B-*aad*A2	*bla*_CTX-M-9_-*ins*B	A
	5	*aad*B-*aad*A2	*qnr*A1- *amp*R	B
	1	*aac*A4-*arr-*3-*dfr*A27- aadA16	short chain dehydrogenase/reductase- *qnr*B6	G
	1	*aac*A4-*bla*_OXA-101_-*cat*B3-*arr*-3	*qnr*A1- *amp*R	K
*Enterobacter aerogenes*	2	*aac*A4-*arr*-3-*dfr*A27- *aad*A16	short chain dehydrogenase/reductase- *qnr*B6	G
*Klebsiella pneumoniae*	1	*aad*B-*aad*A2	*qnr*A1- *amp*R	B
	2	*dfr*A25	*sap*A-like-*qnr*B2	D
	5	*dfr*A12-*orf*F-*aad*A2	*sap*A-like-*qnr*B2	E
	9	*aac*A4-*arr*-3-*dfr*A27- *aad*A16	*sap*A-like-*qnr*B2	F
	15	*aac*A4-*arr*-3-*dfr*A27- *aad*A16	short chain dehydrogenase/reductase- *qnr*B6	G
*Klebsiella oxytoca*	1	*aac*A4-*arr*-3-*dfr*A27- *aad*A16	short chain dehydrogenase/reductase- *qnr*B6	G
*Proteus mirabilis*	1	*bla*_PSE-1_	*dfr*A10	L
*Acinetobacter spp.*	5	*aad*B-*aad*A2	*qnr*A1- *amp*R	B
	3	*cat*B3-*qnr*VC-like-*aac*A4	*bla*_PER-1_-GST-novel type ABC transporter	H
	1	*cat*B-like-*aad*B-*aad*A24-like	*bla*_PER-1_-GST-novel type ABC transporter	J
*Pseudomonas. aeruginosa*	2	*aad*B-*aad*A2	*qnr*A1- *amp*R	B
	1	*aac*A4-like-*bla*_OXA-101_-*aad*A5	*bla*_PER-1_-GST-novel type ABC transporter	I
*Stenotrophomonas. maltophilia*	1	*aad*B-*aad*A2	*qnr*A1- *amp*R	B

This structure which is usually called “complex class 1 integrons” or “complex *sul1*-type integrons”, [4] is the large genetic element in which different class 1 integrons is associated with IS*CR1*. These elements are known to be associated with many resistance genes, encoding resistance to chloramphenicol, trimethoprim, quinolone, and β-lactam, [5] and have two notable structures. Besides, this structure is a powerful gene-capturing tool kit that can mobilize antibiotic resistance genes. The most of their structure comprises a typical class 1 integron with a 5′ conserved segment (5′-CS), a 3′-CS, and an intervening variable region (VR1), followed by a copy of IS*CR1* and then by an IS*CR1*-linked resistance gene region (VR2), which accommodates a variety of resistance genes. This region is, in turn, followed by a repetition of the 3′-CS [6, 7].

## Methods

According to the previous study, sixty three isolates simultaneously harbored class 1 integrons and IS*CR1*-linked resistance genes were selected for further analysis of the connecting the IS*CR1* and the 3′-CS of the integron.

**Fig. 1:**
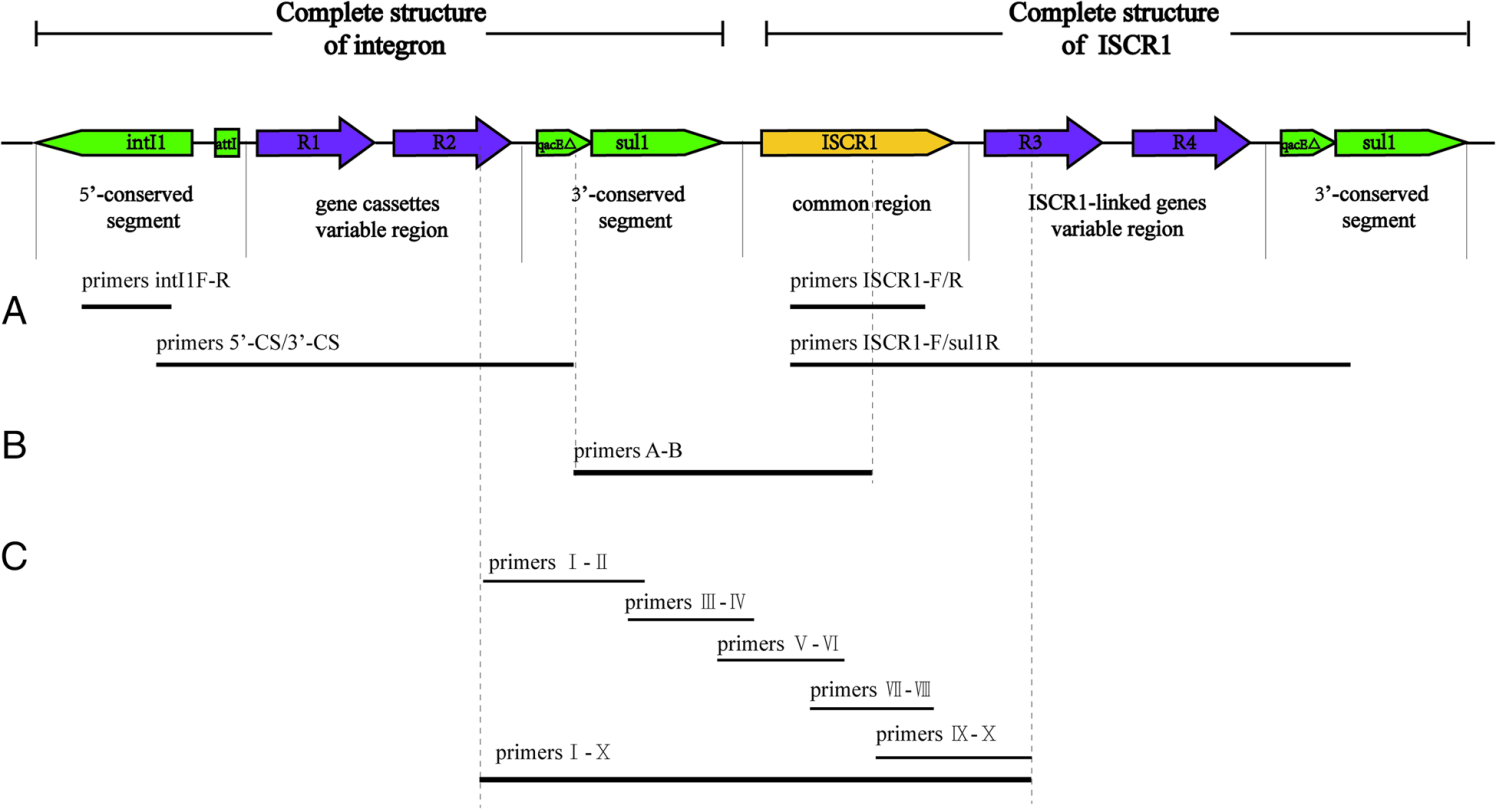
The hypothesis connecting structure between the IS*CR1* and the 3′-CS of the integron. **a** Integrase were amplified by PCR using primer pairs intI1-F and intI-R. class 1 integrons were amplified by 5′CS and 3′CS.IS*CR1* were amplified by PCR using primer pairs IS*CR1*-F and IS*CR1*-R. IS*CR1*-linked genes were amplified by PCR using primer pairs IS*CR1*-F and *sul1*-R. **b** Lengths of the PCR products obtained with primers A and B. **c** Lengths of the PCR products obtained with a series primers (I-X)

According the hypothesis connecting structure (Fig. 1) between the IS*CR1* and the 3′-CS of the integron, primers A and B were designed to amplify the 2045-bp fragment at the junction between IS*CR1* and the 3′-CS of the class 1 integrons and used to preliminarily confirm that IS*CR1* was inserted downstream from the *sul1* gene in the class 1 integrons. Primers I and X, which are specific for the VR1 and VR2 resistance genes investigated in this study, were used to identify the connecting regions: downstream of class 1 integrons and upstream of IS*CR1*. The amplification products of primers I and X were used as the templates in subsequent PCR analyses. Five set primers (Fig. 1) were used to verify this region with the IS*CR1* and the class 1 integrons connected in series.

Primers of the connection region of integron and IS*CR1* are listed in Table 2 and were synthesized by Beijing Genomics Institute (Shenzhen, China). PCR amplifications were performed using 1.5 μL of template, 2 μL of 10 × PCR buffer, 4 μM of each primer stock solution, 4 mM of each dNTP, 1 U of Ex Taq DNA polymerase (TaKaRa Bio Inc., Tokyo, Japan), and sterile distilled water added to a final total volume of 20 μL. Amplification was performed using a Mastercycler® PCR System (Eppendorf International, Hamburg, Germany). Thermocycling parameters were 94 °C for 5 min, 35 cycles of 94 °C for 30 s, 55–60 °C for 5 min, and 72 °C for 2–5 min; a final extension step at 72 °C was added for 5 min. Different annealing temperature and extension of time depended on different length of the PCR amplicons.

**Table 2:** Primers for PCR amplification of the connection region of integron and IS*CR1*

Type of complex class 1 integron	No.	Primer name	Sequence (5′–3′)
A	I	*aad*A2 + *bla*_CTX-9_F	CGTTGCCTTGGTAGGTCC
	II	*aad*A2 + *bla*_CTX-9_ up R	TTGCTTGCCATAGTCATCTT
	IX	*aad*A2 + *bla*_CTX-9_ down F	CCCCAAGGAGCCCATTC
	X	*aad*A2 + *bla*_CTX-9_R	GGTATTCAGCGTAGGTTCAGT
B	I	*aad*A2 + *qnr*A1 F	GTTGTCCCGCATTTGGT
	II	*aad*A2 + *qnr*A1 up R	GGTTGAGCGAGAAGGTTTT
	IX	*aad*A2 + *qnr*A1 down F	GCGTGAGCTGCCACCAGAA
	X	*aad*A2 + *qnr*A1 R	TCTTATGGCTGACTTGATTGTAG
C	I	*aad*A1 + *bla*_DHA-1_ F	ATCTGGCTATCTTGCTGAC
	II	*aad*A1 + *bla*_DHA-1_ up R	TTCCGAGAAGGTGATTGC
	IX	*aad*A1 + *bla*_DHA-1_ down F	CCAACACTGCTCAACACT
	X	*aad*A1 + *bla*_DHA-1_ R	GGTGGCGATTGTGATTCT
D	I	*dfr*A25+ *sapA* F	ACGAAGCGATGGGTAGA
	II	*dfr*A25+ *sapA* up R	AGCCCTCACGAGTTGTTAT
	IX	*dfr*A25+ *sapA* down F	CAAGAAGCCCGACAAAT
	X	*dfr*A25+ *sapA* R	TGGGAGGTGCTGGATAA
E	I	*aad*A2+ *sapA* F	CGTTGCCTTGGTAGGTC
	II	*aad*A2+ *sapA* up R	AACCGCACAATCTCGTC
	IX	*aad*A2+ *sapA* down F	CGCTGCTGATAGACGAAG
	X	*aad*A2+ *sap*A R	TGGGAGGTGCTGGATAA
F	I	*aad*A16+ *sap*A F	GTTGTTCCTTGGCGTTATC
	II	*aad*A16+ *sap*A up R	TCAGCAATATCGGGATAGAG
	IX	*aad*A16+ *sap*A down F	AGACGATACGCTGACTCA
	X	*aad*A16+ *sap*A R	ATGACCGACTGCTTGATG
G	I	*dfr*A27 + short chain F	GCAATGAGGGAGCTAAAGA
	II	*dfr*A27 + short chain up R	TTGGGTTCAGGGTGCTAT
	IX	*dfr*A27 + short chain down F	CAAGAAGCCCGACAAATC
	X	*dfr*A27 + short chain R	TTCACGAGCATAGGCAATA
H	I	*aac*A4 + *bla*_PER_ F	CCCGAGGTCACCAAGA
	II	*aac*A4 + *bla*_PER_up R	GCACCATCCCACATAAGA
	IX	*aac*A4 + *bla*_PER_down F	AAGAGGGCGAAGACGA
	X	*aac*A4 + *bla*_PER_ R	TCCATCAGGCAACAGAAT
I	I	*aad*A5 + *bla*_PER_ F	ACTGGTCTCATTGCTCCTA
	II	*aad*A5 + *bla*_PER_ up R	CGAAGAACCGCACAATCT
	IX	*aad*A5 + *bla*_PER_ down F	CAACACTGCTCAACACTG
	X	*aad*A5 + *bla*_PER_ R	ATTGGTTCGGCTTGACTC
J	I	*aad*A24 + *bla*_PER_ F	CATCATTCCGTGGCGTTA
	II	*aad*A24 + *bla*_PER_ up R	GACACCGAGACCAATAGC
	IX	*aad*A24 + *bla*_PER_ down F	AATCCAACACTGCTCAACA
	X	*aad*A24 + *bla*_PER_ R	CATCATTCCGTGGCGTTA
K	I	*arr*-3 + *qnr*A1 F	GGTAATCCAACACAGTCCTA
	II	*arr*-3 + *qnr*A1 up R	GTCCGCCTCAGCAATATC
	IX	*arr*-3 + *qnr*A1 down F	TCCAACACTGCTCAACAC
	X	*arr*-3 + *qnr*A1 R	CCAGAGTATCCGCAATCC
L	I	*bla*_PSE-1_ + *dfr*A10 F	TTATGGCGGCGTTAGATG
	II	*bla*_PSE-1_ + *dfr*A10 up R	CGAGACCAATAGCGGAAG
	IX	*bla*_PSE-1_ + *dfr*A10 down F	ATATTGAAGTCTGCGAACAC
	X	*bla*_PSE-1_ + *dfr*A10 R	CGTGCTCTGTGATAGTTGA
-	III	common 1 791 F	TATTGCTGAGGCGGACTG
	IV	common 1 791 R	CATTGGAGGAGGTCGTTG
	V	common 2 1054 F	GGCTTCCGCTATTGGTC
	VI	common 2 1054 R	TTGCTTGCCATAGTCATCTT
	VII	common 3 1727 F	TCGCCCACTCAAACAAA
	VIII	common 3 1727 R	GCTCCTCATCCGAAGTATCT
	A	-	CCTGTCGGTGTTGCTTAT
	B	-	GTTGCTTGCCATAGTCATC

**Fig. 2:**
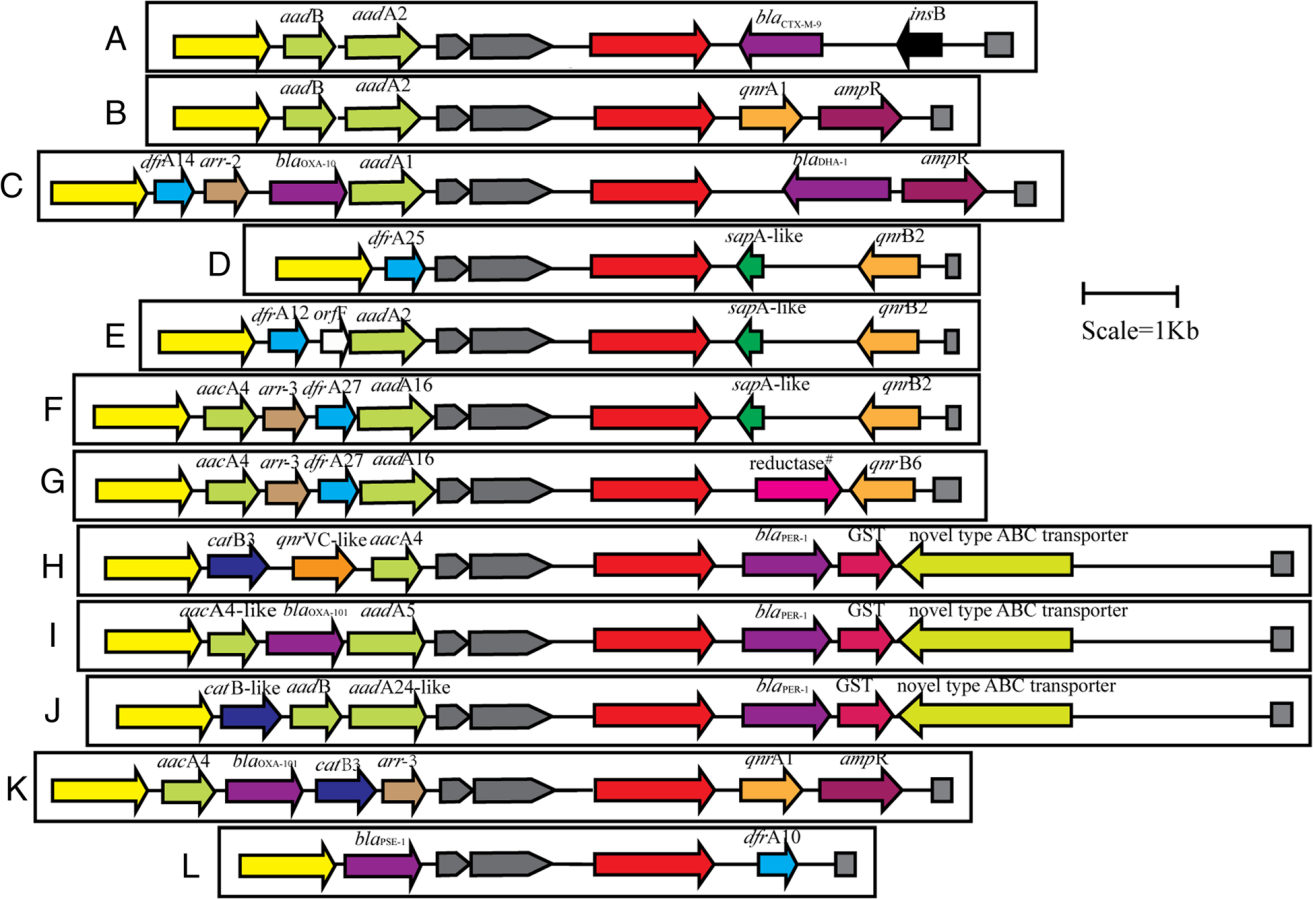
Twelve distinct structures connecting the variable regions of the class 1 integron structures and the IS*CR1*-linked resistance genes in MDR Gram-negative bacteria. The types of the structures were marked from A to L. IS*CR1* is represented by red boxes; integrase is represented by yellow boxes; the *qacEΔ1*/*sul1* is denoted by gray boxes; Open reading frames are indicated with open boxes having their own individual color and the direction of their transcription is indicated with arrows

Types of complex class 1 integron correspond to Fig. 2;

Primers No. correspond to Fig. 1

The PCR amplicons were purified and sequenced (Sanger capillary sequencing) at the Beijing Genomics Institute (Shenzhen, China). The resulting DNA sequences were analysed with the BLAST program at the NCBI homepage (http://www.ncbi.nlm.nih.gov/blast/).

## Results

Thirteen genes (*qnrA1, qnrB2, qnrB6*, *ampR*, *dfrA10*, *bla*_DHA-1_, *bla*_CTX-M-9_, *bla*_PER-1_, *insB*, *sapA*-like, *gst*, and those encoding a novel ABC transporter and a short-chain dehydrogenase/reductase) were detected in the IS*CR1*-linked resistance gene arrays. The gene cassettes of the class 1 integrons found in the 63 isolates included those encoding resistance to trimethoprim (*dfrA12*, *dfrA25*, *dfrA27*), aminoglycosides (*aadA2*, *aadA16*, *aadB*, *aac(6*′*)-Ib*, *aacA4*), chloramphenicol (*catB3*, *catB8*), quinolone (*qnrVC-like*), and rifampicin (*arr-3*, *arr-2*).

**Fig. 3:**
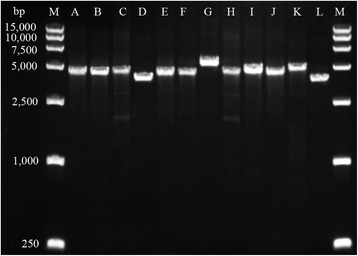
Agarose gel electrophoresis of PCR products obtained with primers I and X for 12 isolates. primers I and X and the region correspond to Fig. 1. The following types corresponding to the Fig. 2 were showed by lane: 1, marker; 2 A; 3, B; 4, C; 5, D; 6, E; 7, F; 8, G; 9, H; 10, I; 11, J; 12, K; 13, L; 14, marker

In this study, the structures (the variable regions of the class 1 integron structures and the IS*CR1*-linked resistance genes) are connected in series among 63 isolates. We found 12 distinct structures connecting the IS*CR1* and the class 1 integron, with a different gene-cassette variable regions, composed of the 5′-CS and the 3′-CS but displaying another unique variable region located between IS*CR1* and the second copy of the 3′-CS (Fig. 2). The PCR products amplified from the regions connecting the first 3′-CS and IS*CR1* are shown in Fig. 3.

## Discussion

To the best of our knowledge, this is the first study to investigate the structure connecting the IS*CR1* and the 3′-CS of the integron in different species in clinical isolates of MDR Gram-negative bacteria on a large scale. It was found that 63 isolates simultaneously carried class 1 integrons and IS*CR1*. The variable regions of the class 1 integron structures to the IS*CR1*-linked resistance genes were linked successfully using long-extension PCR, suggesting that these two structures are connected in series.

Of the 12 type structures, 8 types (A, C, F, H, I, J, K and L type) were first found in any species according to the systematic search in PubMed and GenBank. The structure type K is similar to a structure In37::IS*CR1*::*qnrA1* (accession No. AY259086) [8]. A genetic structure (type B), [*aadB + aadA2*]:IS*CR1*:[*qnrA1 + ampR*], is the same as a structure already reported In293::IS*CR1*::*qnrA1* in *E. cloacae*, [9] which was also found in *E. coli*, *K. pneumoniae*, *Acinetobacter spp*., *P. aeruginosa* and *S. maltophilia* in this study. We detected the structure D type, [*dfrA25*]:IS*CR1*:[*sapA-like + qnrB2*], that was previously found and described in *Salmonella* isolates [10], but which has never before been described in *E. coli* or *K. pneumoniae*. The structure G has been detected in clinical *K. pneumoniae* (accession No. JF775516) isolates in a previous study [11] but was first detected in *E. coli*, *E. cloacae*, *E. aerogenes* and *K. oxytoca*. Besides, the structure G was found in about 32 % isolates (20/63) in this study. The structure E type was previously reported by Ziyong Zong et al. in 2010 from China [12] (accession No. NG037697).

Among the 12 type structures, the VR-2 was similar as previous surveys, whereas the VR-1 was very different from previous surveys. Although the array of gene cassettes in the VR-1 can be easily exchanged, the distribution of IS*CR1*::*qnrB2* in our survey revealed the presence of structure D, E and F. Worldwide, different VR-1 arrays in the structures carrying IS*CR1*::*qnrB2* clinical isolates have been reported eg. In2, In27, In54, In73, In207 and In585 [13]. About structure H, I and J, Ruirui Xia et al. described similar structures carrying *bla*_PER-1_ and *qnrVC*-like genes and made an exhaustive study [14].

Enterobacteriaceae strains carrying the structures are becoming more common [15–17]. In this study, nine distinct structures were identified among Enterobacteriaceae strains, including seven distinct structures connecting the IS*CR1* and the class 1 integron that have never been described in any species. It should be noted that only four distinct structures were identified in MDR non-fermenting isolates. One possible explanation is that chromosomal resistance mechanisms, such as efflux pumps, are more common than laterally transferred genetic resistance factors in these genera in this bacterial population.

## Conclusions

This is the first study to describe the structure connecting the IS*CR1* and the class 1 integron in clinical MDR Gram-negative bacterial isolates in a large-scale study. In total, 12 distinct structures were described. Several types of these structures are similar with the structure of other reports, but not entirely same. This structure is a powerful gene-capturing tool that can mobilize antibiotic-resistance genes. Therefore, the structural analysis of the structure connecting the IS*CR1* and the class 1 integron could guide treatment strategies and provide directions for future research into the mechanisms of bacterial antibiotic resistance.

## Nucleotide sequence accession number

The nucleotide sequences of the structure A to L in this work have been submitted to the GenBank database and assigned accession No. JX880393, JX880383, KM111274, JX880388, KM111278, KM111276, KM111280, JX880386, KM111273, KM111272, KM111271 and KM111275.

## References

[1] Toleman MA, Bennett PM, Walsh TR (2006). ISCR elements: novel gene-capturing systems of the 21st century?. Microbiol Mol Biol Rev.

[2] Wang F, Wu K, Sun J, Wang Q, Chen Q, Yu S, Rui Y (2012). Novel IS*CR1*-linked resistance genes found in multidrug-resistant Gram-negative bacteria in southern China. Int J Antimicrob Agents.

[3] Wu K, Wang F, Sun J, Wang Q, Chen Q, Yu S, Rui Y (2012). Class 1 integron gene cassettes in multidrug-resistant Gram-negative bacteria in southern China. Int J Antimicrob Agents.

[4] Mazel D (2006). Integrons: agents of bacterial evolution. Nat Rev Microbiol.

[5] Toleman MA, Walsh TR (2011). Combinatorial events of insertion sequences and ICE in Gram-negative bacteria. FEMS Microbiol Rev.

[6] Bennett PM (2008). Plasmid encoded antibiotic resistance: acquisition and transfer of antibiotic resistance genes in bacteria. Br J Pharmacol.

[7] Partridge SR, Hall RM (2003). In34, a Complex In5 Family Class 1 Integron Containing orf513 and dfrA10. Antimicrob Agents Chemother.

[8] Wang M, Tran JH, Jacoby GA, Zhang Y, Wang F, Hooper DC (2003). Plasmid-mediated quinolone resistance in clinical isolates of Escherichia coli from Shanghai, China. Antimicrob Agents Chemother.

[9] Bado I, Cordeiro NF, Robino L, Garcia-Fulgueiras V, Seija V, Bazet C, Gutkind G, Ayala JA, Vignoli R (2010). Detection of class 1 and 2 integrons, extended-spectrum beta-lactamases and qnr alleles in enterobacterial isolates from the digestive tract of Intensive Care Unit inpatients. Int J Antimicrob Agents.

[10] Wu JJ, Ko WC, Chiou CS, Chen HM, Wang LR, Yan JJ (2008). Emergence of Qnr determinants in human Salmonella isolates in Taiwan. J Antimicrob Chemother.

[11] Ruiz E, Saenz Y, Zarazaga M, Rocha-Gracia R, Martinez-Martinez L, Arlet G, Torres C (2012). qnr, aac(6′)-Ib-cr and qepA genes in Escherichia coli and Klebsiella spp.: genetic environments and plasmid and chromosomal location. J Antimicrob Chemother.

[12] Zong Z, Partridge SR, Iredell JR (2010). ISEcp1-mediated transposition and homologous recombination can explain the context of bla(CTX-M-62) linked to qnrB2. Antimicrob Agents Chemother.

[13] Quiroga MP, Arduino SM, Merkier AK, Quiroga C, Petroni A, Roy PH, Centron D, Argentinian Integron Study G (2013). “Distribution and functional identification of complex class 1 integrons”. Infect Genet Evol.

[14] Xia R, Guo X, Zhang Y, Xu H (2010). qnrVC-like gene located in a novel complex class 1 integron harboring the IS*CR1* element in an Aeromonas punctata strain from an aquatic environment in Shandong Province, China. Antimicrob Agents Chemother.

[15] Richter SN, Frasson I, Bergo C, Manganelli R, Cavallaro A, Palu G (2010). Characterisation of qnr plasmid-mediated quinolone resistance in Enterobacteriaceae from Italy: association of the qnrB19 allele with the integron element IS*CR1* in Escherichia coli. Int J Antimicrob Agents.

[16] Santos C, Caetano T, Ferreira S, Ramalheira E, Mendo S (2011). A novel complex class 1 integron found in a Klebsiella pneumoniae isolate from Portugal. Clin Microbiol Infect.

[17] Sun C, Su Z, Zhou C, Liu Y, Yuan H, Yin J, Xu H (2012). Complex class 1 integron containing bla (CTX-M-1) genes isolated from Escherichia coli: a potentially novel resistant gene-capturing tool kit. Curr Microbiol.

